# The Spectrum of Clinical and Pathological Manifestations of AIDS in a Consecutive Series of 236 Autopsied Cases in Mumbai, India

**DOI:** 10.4061/2011/547618

**Published:** 2011-05-23

**Authors:** Dhaneshwar Namdeorao Lanjewar

**Affiliations:** Sir J. J. Hospital and Grant Medical College, Byculla, Mumbai, Maharashtra 400008, India

## Abstract

The HIV epidemic in the Asian subcontinent has a significant impact on India. The AIDS associated pathology has not been well evaluated in a representative study hence very little is known about the spectrum of HIV/AIDS associated diseases in Indian subcontinent. To determine the important postmortem findings in HIV infected individuals in Mumbai, autopsy study was carried out. The patient population included patients with AIDS who died at the tertiary care hospital over a 20 year period from 1988 to 2007. A total of 236 (182; 77% males and 54; 23%) females) patients with AIDS were autopsied. The main risk factor for HIV transmission was heterosexual contact (226 patients; 96%) and 223/236 (94%) patients died of HIV-related diseases. Tuberculosis was the prime cause of death in 149 (63%) patients, followed by bacterial pneumonia 33 (14%), cryptococcosis 18 (8%), toxoplasmosis of brain 15 (6%), pneumocystis jiroveci (PCJ) 1 (0.5%) and Non-Hodgkin's lymphoma 7 (3%) cases. The major underlying pathologies are either preventable or treatable conditions. There is an urgent need for attention towards the diagnosis, issue of therapy, and care of HIV disease in developing countries. Reducing mortality in patients with AIDS from infections must be highest public health policy in India.

## 1. Introduction

India has a population of over 1.19 billion, around half of whom are adults in the sexually active age group. The first AIDS case in India was detected in 1986; since then HIV-infection has been reported in all states and union territories [1]. The spread of HIV in India has been uneven, with much of India having a low rate of infection; certain places have been more affected than others. HIV epidemics are more severe in the southern half of the country and the far North East. The highest HIV prevalence rates are found in Maharashtra in the west, Andhra Pradesh, Tamil Nadu, and Karnataka in the south, and Manipur and Nagaland in the North East. In the Maharashtra and the southern states, HIV is primarily spread through heterosexual contact. Infections in the North East are mainly found amongst injecting drug users and sex workers [2]. Previously it was thought that around 5 million people were living with HIV in India more than in any other country. Better data, including the results of a national household survey conducted in 2005-2006, led to a major revision of the prevalence estimate in July 2007, and it is now thought that around 2.31 million people in India are living with HIV [2]. Of these, an estimated 57.5% are male, 39% female, and 3.5% are children. Across India HIV prevalence appears to be low (0.34%) in the general population, but disproportionately high among high-risk groups, such as injecting drug users (7.2%), female sex workers (5.1%), men who have sex with men (7.4%) and sexually transmitted disease clinic attendees (3.6%) [2].

Maharashtra is the second largest state in India with population of 100 million. Maharashtra has the highest rate (42%) of urbanization, major national highways pass through the state, and it has a high rate of migrant and floating population. There exists a well-established sex industry in the state. The bed occupancy in many hospitals due to HIV-positive persons has been as high as 25% to 30%. Mumbai is the most populous city in the Maharashtra state of India with an estimated population of 13.7 million. The HIV/AIDS scenario in Mumbai is alarming. HIV-positive rates in various categories in Mumbai are among women attending antenatal clinics (1.2%), sexually transmitted disease clinic attendees (27.4%), female sex workers (42.4%), men having sex with men (8.4%), Intravenous drug users (24.4%), Eunuchs (42.1%), and in voluntary HIV testing centers (10.7%) [3]. Studies on selected group of HIV-positive adults in India have shown that tuberculosis (TB) is major opportunistic infection and that Pneumocystis jiroveci (PCJ) pneumonia is an uncommon cause of pulmonary symptoms [4–7]. The Indian literature on HIV/AIDS describes few reports regarding relative prevalence of opportunistic infections, tumors, and other HIV-associated pathologies [7–31]. 

## 2. Materials and Methods

Sir J. J. Hospital in Mumbai is a public hospital run by the State Government that provides free services to patients and also treats all patients with HIV-associated diseases. The study population was adults (aged more than 18 years) seen at Sir J. J. Hospital (the same hospital referred to in previous work) [7, 11, 13–16, 18, 24, 31]. These patients were admitted in medical wards (internal medicine, pulmonary medicine, emergency medicine, and dermatology) of a 1300-bed hospital. The patients admitted to neurology or neurosurgery wards were not included in this study. Consecutive adults admitted to medical wards were questioned and examined for signs and symptoms of HIV-infection and their blood had been taken for HIV antibody testing. Antibodies to HIV-1 and HIV-2 were detected using DETECT-HIV enzyme linked immunosorbent assay (Biochem Immunosystem, Inc., Montreal, Canada). All the sera reactive by above test kit were further tested using Immunocomb HIV-1 and HIV-2 Bispot test kit (Organics, Yavne, Israel). The sera that were reactive by both test systems were labeled as HIV-reactive sera and the patients were determined to be HIV-positive. All the cases included in this study were reactive for HIV-1 antibodies and none showed HIV-2 reactivity. The facilities for CD4 and CD8 testing were not available in our hospital until June 2007 and patients were unable to afford the cost of the CD4 and CD8 testing from private laboratories. In July 2007, CD4 and CD8 testing facilities were made available to our patients. In April 2004, Government of India launched a program of providing free HAART at the eight Government hospitals (including Sir J. J. Hospital, Mumbai) in the country. Only two patients in the present study had received HAART therapy through this program. 

Mumbai city was the only one in India where Coroner's Act was in place, requiring all deaths (including hospital deaths) to be reported to the Coroner's office. This office also gave permission for postmortem examinations; and approximately 30% of the hospital deaths in Sir J. J. Hospital, Mumbai were subjected to postmortem examination. Since the abolition of the Coroner's Act in July 2000, postmortem examination was carried out after obtaining consent from deceased's next of kin. Postmortem examination in 140 out of 236 cases (59%) was carried out between 1988 to 2000 when Coroner's Act was existing while postmortem in 96/236 (41%) cases were carried out after Coroner's act was abolished. All the cases in the present study were consecutive autopsies without any selection bias. Complete autopsies on all cases were performed, including removal of brain (but not spinal cord or eyes).

The main risks associated with performing an autopsy on HIV-infected individuals stem from contact with blood or body fluids, penetrating injuries and aerosols. HIV-infected patients often have multiple opportunistic and other infections with the potential for transmission to staff in mortuary. Therefore while performing autopsies in HIV-infected cases standard guidelines were followed [32]. Protective clothing such as surgical clothing, plastic body suits with long sleeves, face mask and glasses, two pairs of latex gloves, and plastic boot with shoe cover were used for each autopsy. The instruments used were kept to a minimum, and blunt ended instruments were used in preference to sharp-pointed instruments. En masse technique based on method originally described by Letulle was adopted for performing HIV necropsies [33]. After removing organ bloc 1 centimeter thick slices of all the internal organs were made and, the organ blocs were preserved in 10% formal saline for at least 24 hours. The formalin-fixed organ bloc was dissected and detailed description of macroscopic findings was documented. Irrespective of presence or absence of grossly visible lesion a minimum of 5 to a maximum of 10 tissue samples were obtained from each major organ which were further submitted for paraffin embedding and 4 micron thick tissue sections were obtained for histopathological assessment. The slides were stained with Haematoxylin and Eosin, Periodic Acid Schiff, Mucicarmine, Ziehl-Neelsen, Gram stain, and Gomori's Methenamine silver nitrate stain. In eight cases immunohistochemistry was used to evaluate lymphoma. At autopsy no cultures for bacteria, fungi, or viruses were obtained. 

## 3. Results

**Figure 1:**
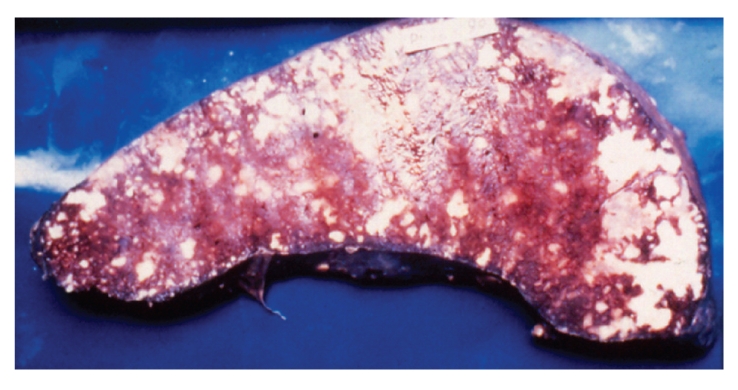
Cut surface of spleen shows tubercles and large areas of caseous necrosis.

**Figure 2:**
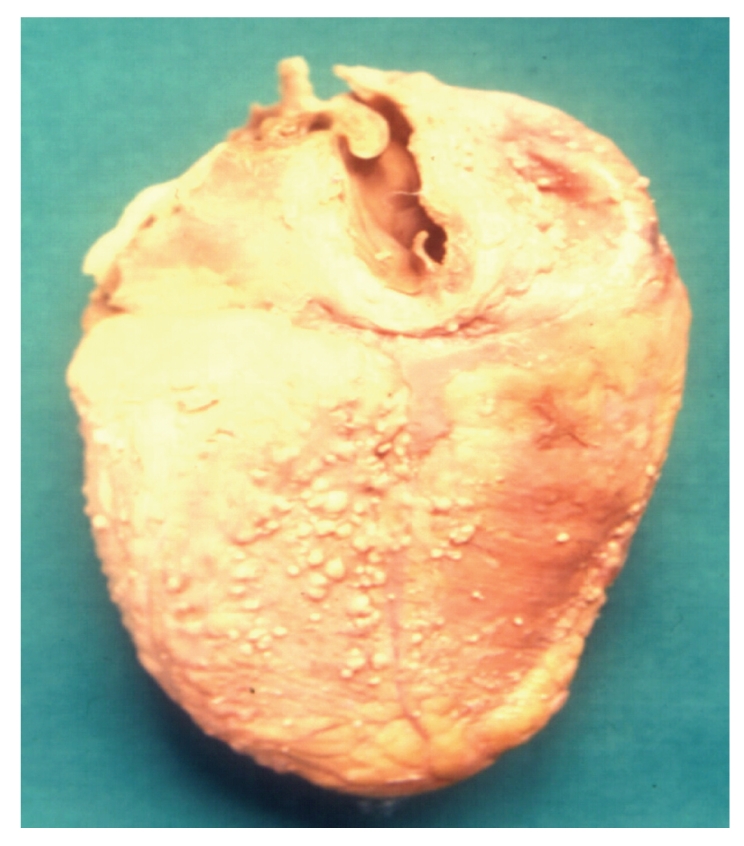
Pericardial surface of heart shows tubercles.

**Figure 3:**
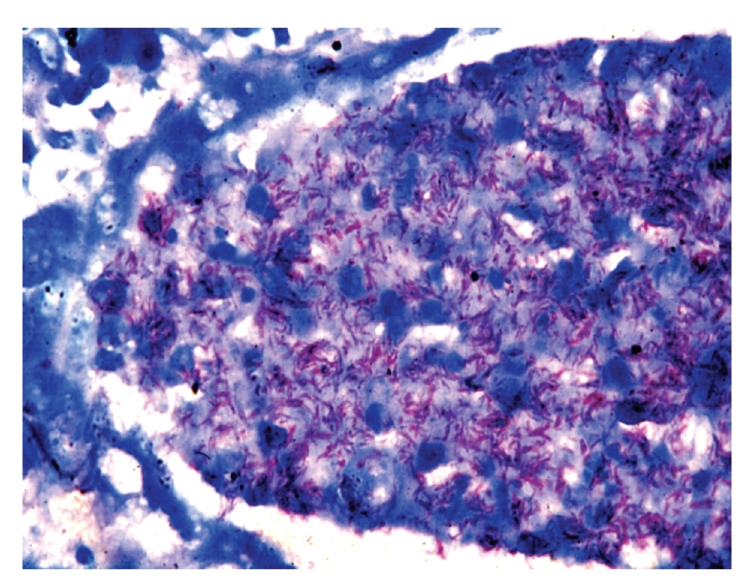
Microscopy of kidney shows tubercle bacilli in tubular lumen (Ziehl-Neelsen stain ×1000).

Demographic data for 236 patients with AIDS shows that a total of 182 (77%) men and 54 (23%) women were included in the study (Table 1). The majority of the patients (192; 81%) were aged 21–40 years. The main risk factor for HIV transmission was heterosexual contact (226 patients; 96%). Other risk factors for HIV transmission were blood transfusion (4 patients; 2%), men having sex with men (3 patients; 1%), and unknown risk factor (3 patients; 1%). Of the 236 HIV-positive patients autopsied 223 (94%) died of AIDS-associated pathologies while death in remaining 13 (6%) cases was due to non-AIDS associated diseases. 150 out of 223 (67%) cases had one, 69 (31%) cases had two, and 4 (2%) cases had three pathologies, thus a total of 300 lesions were identified in 223 cases. Discrepancy between antemortem and postmortem diagnosis was found in 42% cases. The prime causes of death and specific AIDS pathology prevalence in HIV positive patients are shown in Table 2. Tuberculosis was the prime cause of death in 149 (63%) patients. Of the 223 patients with AIDS defining pathology 152 (68%) had tuberculosis. 60% (91 out of 152) patients of tuberculosis had hepatosplenomegaly, 50% (76 out of 152), cervical and/or axillary lymphadenopathy, 43% (65 out of 152) weight loss, 25% (38 out of 152) chronic diarrhea, and 22% (34 out of 152) had recurrent fever. TB was widely disseminated in 143 (94%) patients, while 9 (6%) patients showed isolated miliary tuberculosis of lung. Organs involved in cases with disseminated tuberculosis were, lymph node 131 (59%), spleen 127 (57%) (Figure 1), liver 118 (53%), kidney 87 (39%), brain 29 (13%) (TB meningitis 28 and tuberculoma 1), adrenal 18 (8%), GIT 11 (5%), heart 9 (4%), (Figure 2), thyroid 14 (6%), pancreas 6 (3%), and prostate 2 (1%). The predominant histological pattern of HIV-associated tuberculosis was non-reactive, abundant granular necrosis, ill formed or absent granulomas, scanty or no giant cells, scanty or no epithelioid cells and numerous acid-fast bacilli on a Ziehl-Neelsen stain (Figure 3). No premortem or postmortem cultures for tubercle bacilli were obtained and a diagnosis of tuberculosis was established only on histology alone, hence all these cases are diagnosed as “presumptive tuberculosis.”

**Figure 4:**
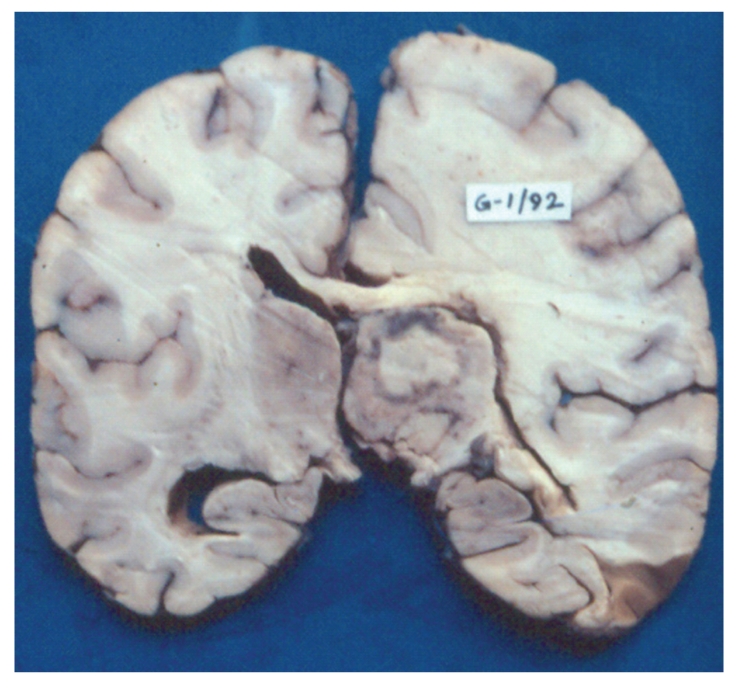
Coronal section of cerebrum shows well circumscribed areas of necrosis surrounded in thalamus.

**Figure 5:**
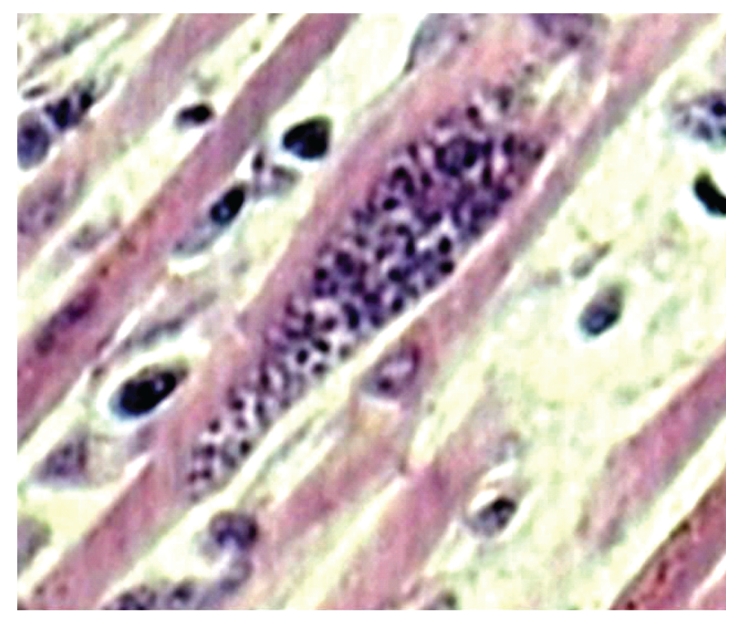
Microscopy of heart shows pseudocyst of toxoplasma gondii in myocardial muscle fiber (H & E ×300).

Bacterial pneumonia was the prime cause of death in 33 (14%) cases (lobar pneumonia; 13 cases and bronchial pneumonia: 20 cases). Clinically patients with bacterial pneumonia presented with breathlessness, fever, and weight loss. Histology of lungs in these patients showed Gram positive cocci (staphylococci and streptococci or both) in 27, Gram negative bacilli in 3, mixed Gram positive cocci, and Gram negative bacilli in 3, none were cultured. Minor lesions of bronchopneumonia were noted in additional 15 cases. Cryptococcosis was found in 18 (8%) patients and was the cause of death in all the cases. In 17 cases, cryptococcal meningoencephalitis was associated with wide spread visceral organ involvement while 1 case showed isolated cryptococcal meningitis. Cerebral toxoplasmosis was the fourth most common cause of death and was noted in 15 out of 223 (7%) cases. All cases with CNS toxoplasmosis were in the age range of 23 to 35 years. The ante mortem signs and symptoms of these cases included convulsions (10 cases), abnormal behavior (5 cases), and inability to walk (5 cases), drowsiness (4 cases) and slurred speech (2 cases). The clinical diagnosis offered in cases with CNS toxoplasmosis was TB meningitis (6 cases), tuberculoma brain (6 cases), pulmonary TB with HIV-infection (1 case), cerebrovascular accident with HIV infection (1 case), and HIV encephalopathy (1 case). None of the cases with CNS toxoplasmosis were suspected clinically. All the patients with CNS toxoplasmosis received antituberculous drugs; there was no improvement in clinical signs and symptoms and subsequently all the patients succumbed to death. Morphology of brain in these cases showed circumscribed necrotic lesions in the white matter, gray matter, basal nuclei, brain stem, and cerebellum (Figure 4). The microscopic examination of the brain showed features of diffuse cerebritis and areas of ischemic necrosis. The necrotic areas showed sparse inflammation and presence of pseudocyst, true cyst, and free forms of toxoplasma gondii. The blood vessels showed vasculitis with or without thrombi and few vessels showed changes of hypertrophic occlusive arteritis. Other opportunistic infections were not identified in these brains. 2/15 cases of cerebral toxoplasmosis showed cardiac toxoplasmosis (Figure 5). 

**Figure 6:**
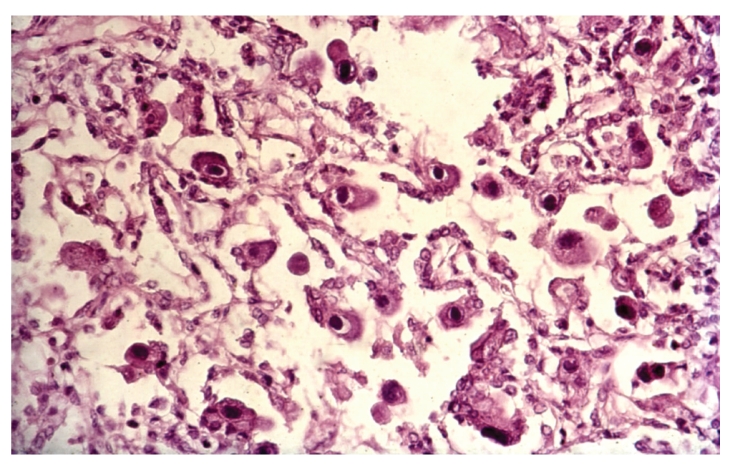
Microscopy of lung shows CMV infection (H & E ×200).

**Figure 7:**
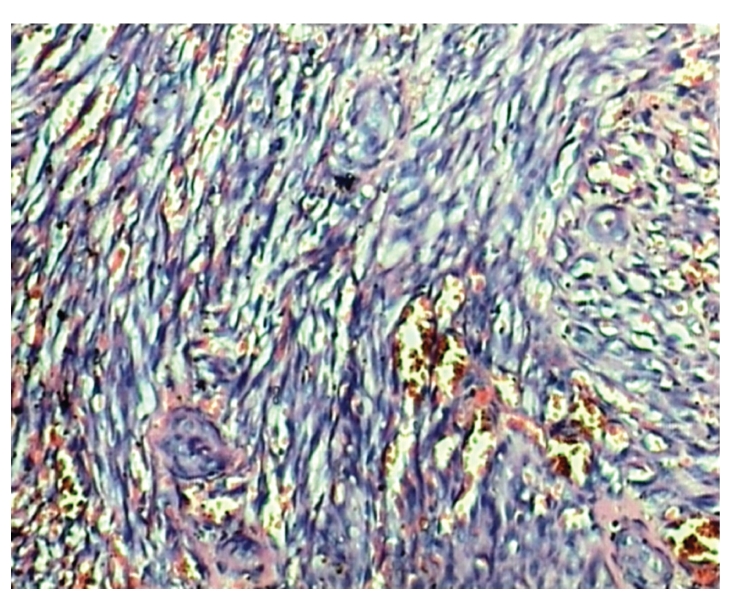
Microscopy shows spindle cells and slit like vascular channels containing erythrocytes (H & E ×400).

 PCJ was identified in 11 (5%) patients. In only one case PCJ was cause of death, while in another 10 cases PCJ was identified in association with tuberculosis, cryptococcosis, and CMV infection. Extra pulmonary PCJ was not present in any patients. Cytomegalovirus infection was identified in 35 (16%) patients. Infection by CMV showed minor lesions, and it was contributory cause of death in these patients. CMV infection was identified in GIT (15), adrenal (8), lung (7), thyroid (4), pancreas (2), kidney (1), heart (1), and brain (1) (Figure 6). Cryptosporidial enteritis was identified in 6 (3%) cases; and it was contributory cause of death in these patients. None of the patients showed hepatobiliary cryptosporidiosis. Candidiasis was noted in 6 cases (esophagus 5 cases and stomach 1 case). 2/236 cases had Kaposi's sarcoma (1%)) (Figure 7). One case of Kaposi's sarcoma was citizen of Mumbai (India), while another was citizen of Nigeria. Death in both cases of Kaposi's sarcoma was due to wide spread tuberculosis. The lesions of Kaposi's sarcoma were not severe and disseminated sufficient to be the cause of death. Additional infectious diseases (neither contributory nor prime cause of death) identified in this study were aspergillosis of lung (6 cases, 3%) and intestinal strongyloidiasis (3 cases, 1%). Lymphoma was found in eight (3%) cases; in seven out of eight cases lymphoma was cause of death; one case of primary hepatic lymphoma died due to widespread cryptococcosis. All were men and 7 out of eight cases showed features of diffuse, large B-cell, non-Hodgkin's lymphoma. The immunohistochemical study in these cases showed positivity for LCA, CD34, and CD68, and negativity for CD3, P53, and Bcl2. One case of primary non-hodgkin's lymphoma of liver (diffuse large T-cell type) showed large polygonal cells with a deeply eosinophilic cytoplasm and large nuclei and immunohistochemical analysis of it showed the lesion to be homogeneously positive for CD3, CD4, CD8, and CD43. In the present study 13/236 (6%) patients died due to non-AIDS-associated pathologies. In six (3%) patients cause of death was cirrhosis of liver. Three patients of cirrhosis showed abnormal liver function tests and presence of HBsAg in serum, while another three patients had history of chronic alcoholism, these cases showed features of alcoholic cirrhosis. The other prime causes of deaths were pulmonary hemorrhage (1 case), cerebral malaria (1 case) pyogenic meningitis (1 case), bacterial peritonitis (1 case), acute pyelonephritis (1 case), amyloidosis (1 case), and squamous cell carcinoma of lung (1 case). In 12 cases ante mortem diagnosis of malaria was suspected; however autopsy examination in only one case showed death due to cerebral malaria. 

## 4. Discussion

Eunuchs are inhabitant in all parts of India and have high risk behavior which is responsible for HIV transmission and are not identified in industrialized countries and sub-saharan Africa. Eunuchs and their existence are well described in ancient Indian texts [34]. In India Eunuchs (hijras) are seen as “a third gender” which is neither male nor female but contains elements of both. Eunuch is an intersexed impotent man who undergoes emasculation. Originally eunuchs were boys either sold by their poor parents or kidnapped. Subsequently they were castrated, set up as beggars, and used as money making machines. The adult life of a Hijra is a male homosexual prostitute playing a passive recipient role and homosexual prostitution in them is said to be institutionalized [35]. They are likely to have multiple partners, even multiple sexual acts a day; therefore they are more prone to acquire HIV-infection. Participation in ancient traditions is facilitating the current spread of HIV-infection throughout India, and in some of the festival celebrations thousands of pilgrims from across the country engage in acts of unprotected sexual intercourse. Many of the festival participants are hijras, eunuchs, and transsexuals who sell sex for a living. Thus in India some ancient traditions are facilitating the current spread of HIV-infection [36]. In a study from south India 5% of eunuchs have been shown to be HIV positive [37]. A large population of eunuchs live in slums of Mumbai and HIV positivity among eunuch's in Mumbai is 42.1% [3].

The spread of HIV in India is worrisome since India is home to a population of over 1 billion and as a single nation it has more people than the continents of Africa, Australia, and Latin America combined. Autopsy continues to be a means of establishing the diagnosis of previously undetected cases or of confirming the diagnosis in those suspected during life. Despite the advances in diagnostic radiology, laboratory analysis, and endoscopy, autopsy has remained a valuable tool for the identification and understanding of diseases for hundreds of years [38–40]. HIV necropsies provide very interesting cases and the systematic postmortem examination of seropositive adults and children dying in hospitals provide representative data on the main infections and tumors that characterize AIDS in that community [41, 42]. The wealth of macroscopic pathology found at necropsy in most patients with AIDS provides excellent material for teaching medical students and postgraduates. With histology, these necropsies make valuable clinicopathological conferences, emphasizing the pathologist's role in the management of HIV disease [40, 42]. The postmortem studies have been fundamental in the early description of AIDS [43–91]. Though AIDS is fundamentally the same disease all over the world, the spectrum of opportunistic infections that occur is governed to a large extent by the endemicity of microorganisms prevalent in the environment. The pattern of opportunistic infections in patients with AIDS in India cannot be predicted from the experience of industrialized countries; however it could be predicted from the experience in Africa. In Africa, to supplement clinical studies of HIV disease systematic post mortem examination of seropositive adults has been carried out by two groups, one at Kinshasa, Zaire, supported by CDC, Atlanta [57] and another at Abidjan, supported by Rockefeller Foundation and Global Program on AIDS, World Health Organization and Medical Research Council, United Kingdom [67]. The present autopsy study was initiated to know the spectrum of pathologic disease processes responsible for deaths in patients with HIV/AIDS in Mumbai, India. With 236 autopsies evaluated, the present study is the largest autopsy study in hospitalized patients with HIV/AIDS in India. 

The initial reports of acquired immunodeficiency syndrome were described in USA in Mid 1981, in homosexual males; these papers described presence of pneumocystis carinii (PCJ) and disseminated Kaposi's sarcoma on postmortem examination [43–46]. Simultaneously several small to large series of autopsy reports are described in the literature [47–55, 57, 61–63, 65–74, 76–78, 80, 81, 83–89, 91–94]. Few autopsy series described organ-specific pathology in patients with AIDS such as pulmonary [7, 75, 82, 90], GIT [11], cardiac [15], renal [16], hepatic pathology [12, 24, 25], and neuropathology [13, 58–60, 79, 93]. One report of needle necropsy in patients with AIDS is described in Indian literature [22]. A comparative chart of various autopsy studies in AIDS, including the present study is shown in Table 3. Only those postmortem studies in which hundred and more autopsies are performed are considered for comparison. The largest autopsy series reported till today was of 565 cases from United States of America [68]; our study represents the largest autopsy report of 236 cases, from India. A complete autopsy is the goal of every postmortem examination; however literature describes a report of needle necropsy in patients with AIDS [22]. The needle necropsy technique is inferior to the conventional autopsy in determining the cause of death [95, 96]. Gross findings of organs cannot be observed by needle necropsy, and this technique cannot detect deep-seated lesions especially in brain and gastrointestinal tract.

The spectrum of diseases identified in our patients differs in several ways from the findings reported in the literature. Infectious diseases were causes of deaths in 216/223 (97%) of our patients. The present data underline the overwhelming significance of TB in HIV disease in India. As the most common cause of death; it was present in 63% of all patients and in 68% of those dying with AIDS defining pathology. Tuberculosis is identified in 5–27% cases in United States [68, 72, 75], in 25% from Mexico [62], in 14% from Brazil [90], in 14–72% from Russia [85, 92], and in 38–54% [67, 78, 81, 94] from Africa. The prevalence of TB identified in our study is similar to studies from Africa and Russia. In the present study 13% of all patients with AIDS defining pathology had TB meningitis or tuberculoma. A high prevalence (10%) of TB meningitis in HIV-infected patients has been reported from Spain [97]. MAI is reported in 13–22% from studies in USA [50, 55, 68, 72, 75, 80], in 6–27% from Brazil [84, 90] and in 39% from Japan [76]. Since culture of postmortem tissues was not carried out we could not document any case of MAI in the present study. The importance of bacterial pneumonia; was documented in one of the earlier autopsy studies in which bacterial infection was identified in 83% cases [98]. Subsequently other studies have shown that bacterial infections occur more frequently than other opportunistic infections in patients with AIDS [51, 55, 67]. Bacterial pneumonias were the second most common pathology in our study which was noted in 21% cases. Bacterial pneumonia is described in 41-42% from United States [75, 80], in 21–30% [67, 81, 94] from Africa, in 36% [90] from Brazil, and in 8% [85] from Mexico. Pyogenic infections are also an important cause of death in patients with AIDS and the findings of the present study support this. We found one case each of pyogenic meningitis, bacterial peritonitis, and acute pyelonephritis. Findings of bacterial meningitis in 2% cases have been observed in few studies [67, 81]. Disseminated cryptococcosis is commonly found in patients with AIDS; in the present study cryptococcosis was identified in 8% cases. Cryptococcosis is described in 8–14% cases from United States [50, 55, 68, 72, 75], in 10% from Mexico [62], in 3–19% from Africa [66, 67], in 3-4% from Brazil [84, 90], and in 14% from Japan [76]. A clinical series from western India (Pune; Maharashtra) reported cryptococcal meningitis in 67% cases [99]. Cerebral toxoplasmosis is the most common cause of cerebral mass lesion in patients with AIDS, and it was found in 7% cases in the present study. CNS toxoplasmosis is described in 3–9% patients from USA [55, 68, 72, 80], in 11–15% from Africa [66, 67], in 7% from Russia [85], in 7–9% from Brazil [84, 90], and in 14% cases from Japan [76]. 

At the onset of AIDS epidemic, PCJ was the most common serious complication, affecting nearly 75% of patients with the syndrome [100]. PCJ is described in 24–60% cases from United States [55, 68, 72, 75, 80], in 24% from Mexico [62], in 27% from Brazil [90], in 35% from Japan [76], in 12% from Russia [92], and in 3–11% from Africa [67, 81]. In the present study PCJ was identified in 5% cases. A low prevalence of PCJ (9%) has also been documented in south India, Africa, and Russia [6, 67, 81, 92]. The incidence of PCJ is also declining as a result of chemoprophylaxis and antiretroviral therapy [101, 102]. Extra pulmonary PCJ is described in lymph node, spleen, kidney, liver, gall bladder, adrenal gland, heart, pancreas, eye, pituitary gland, thyroid gland, lower urinary tract, bone marrow, and skin [68]. No case of extra pulmonary PCJ was identified in the present study. Cytomegalovirus infection is described in 17–69% cases from USA [50, 55, 68, 72, 75, 80], in 74% from Japan [76], in 69% from Mexico [62], in 13–18% from Africa [66, 67], in 6–19% from Russia [85, 92], in 13–18% from Brazil [84, 90], and in 65% from Poland [71]. In the present study CMV was identified in 16% cases, this is similar to reports from Africa, Brazil, and Russia. None of our patients with CMV infection had significant inflammation or necrosis; the lack of an inflammatory response may represent severe immunosuppression in our patients. In the present study candidiasis was identified in 3% cases. Candidiasis is described in 2–88% cases from USA [50, 55, 68, 72, 75], 31% cases from Africa [66], 13% cases from Brazil [84], 37% cases from Japan [76], and 9% cases from Russia [92]. Increased incidence of penicillium marneffei infection is reported in North East State (Manipur) in India [17, 19]. Other than Manipur no reports of this mycosis are documented in Indian literature. Cryptosporidiosis is described in 4% cases from USA [55] and 3% cases from Africa [67]. The 3% prevalence of cryptosporidiosis in the present study is probably underestimation due to rapid autolysis of intestinal mucosa after postmortem examination. Our previous report of investigation of faecal specimens in patients with AIDS presenting with chronic diarrhea showed high prevalence of cryptosporidiosis (13%) and isosporiasis (17%) [103]. 

Kaposi's sarcoma and high grade B-cell non-Hodgkin's lymphoma are the prototypical AIDS-defining malignant diseases. Very little is known regarding the spectrum of neoplasm in Indian patients with AIDS [104–106]. The cancer pattern among HIV positive patients in Mumbai showed NHL in 31% and carcinoma cervix in 13% of cases [104]. This report showing high prevalence of NHL and carcinoma cervix is from largest cancer hospital in the country where cancer patients from all over India are referred and it does not reflect general prevalence of cancer in patients with AIDS. Lymphoma is described in 1–14% cases from USA [50, 55, 68, 75, 77, 80], in 3% cases from Africa [67], in 9% cases from Mexico [62], in 32% cases from Japan [76], in 6% cases from Russia [92], and in 2% cases from Brazil [90]. In the present study lymphoma was noted in 4% cases. A report from south India describes three cases of HIV-associated primary CNS lymphoma [27]. A rare case of HIV-associated primary ovarian lymphoma is also described in Indian literature [107]. The first case of AIDS-associated Kaposi's sarcoma was identified in India in the year 1992 [8]. Since then only eight reports of KS are described in the Indian literature [8, 20, 26, 28, 30, 108, 109]. In the present study only 2 (1%) cases of Kaposi's sarcoma were identified; these cases were published earlier as short reports [8, 30]. Kaposi's sarcoma is described in 8–50% cases from USA [50, 55, 68, 75, 80], in 9–11% cases from Africa [67, 81], in 30% cases from Mexico [62], in 14% cases from Japan [76], in 10% cases from Russia [85], and in 4% cases from Brazil [90]. The low prevalence of Kaposi's sarcoma in India may be explained due to low prevalence of HHV-8 in our population [110]. 

Over the past two decades, autopsies have become increasingly difficult to arrange. A systematic review of autopsy series over the past 40 years has suggested an error rate of up to 24.4% in preautopsy clinical diagnosis [111]. A recent autopsy report on HIV-infected cases showed that primary diagnosis was changed by autopsy in 70% cases [91]. The present study also showed discrepancy between ante mortem and postmortem diagnosis in 42% cases. The autopsy studies in India are dwindling due to phobia on part of pathologists to carry out autopsies on HIV-infected cases. To address this, a group of pathologists discussed an issue of fear during HIV-infected autopsies among mortuary staff (pathologists and technical staff) and after extensive deliberation they unanimously agreed that HIV necropsy work was safe when carried out sensibly [42]. Our institute in Mumbai was the first center in the country to perform postmortems in patients with HIV/AIDS. Subsequently other medical colleges in Maharashtra (Mumbai and Pune) performed postmortems and reported findings in patients with AIDS [10, 21, 22, 25]. However due to fear of likelihood of becoming infected with HIV, pathologists from one center in Pune stopped performing HIV autopsies [21], while pathologists from another center in Pune used an alternate method of performing needle necropsy in HIV-positive patients [22]. With the national policy of providing ART to adults with AIDS, a new cohort of patients is emerging with altered biology of infection confusing the clinical picture. With passage of time, these patients need to be evaluated in the light of new knowledge following specific therapy and compared with the cohort which did not receive antiretroviral therapy. 

## 5. Conclusion

The present study represents the largest autopsy report defining the spectrum of AIDS-associated diseases in the Indian subcontinent. The present data underline the overwhelming prevalence of tuberculosis in HIV-infected patients in Mumbai. It is imperative that major laboratory efforts be directed to identifying tuberculosis and other opportunistic infections. The information on opportunistic infections generated through this study will be useful for the management of HIV-infected patients. This data also underlines that there is unusual paucity of pneumocystis jiroveci and Kaposi's sarcoma in our population. Reducing mortality in patients with AIDS from infections must be highest public health policy in India. 

## Figures and Tables

**Table 1:** Demographic data in HIV/AIDS (*N* = 236).

Age group (years)	Male	Female	Total (%)
18–20	5	3	8 (3%)
21–30	70	21	91 (39%)
31–40	79	22	101 (43%)
41–50	18	4	22 (9%)
>50	10	4	14 (6%)

Total	182 (77%)	54 (23%)	236 (100%)

Risk factors: heterosexual, 226 (96%) cases; blood transfusion, 4 (2%) cases, men having sex with men, 3 (1%) cases, unknown risk factor, 3 (1%) cases.

**Table 2:** The prime cause of death and pathology prevalence in patients with HIV/AIDS.

Diseases	Prime cause of death (*N* = 236)	AIDS pathology prevalence (*N* = 223)
Tuberculosis	149 (63%)	152 (68%)
Bacterial pneumonia	33 (14%)	48 (21%)
Cryptococcosis	18 (8%)	18 (8%)
Toxoplasmosis	15 (6%)	15 (7%)
PCJ	1 (0.5%)	11 (5%)
Non-Hodgkin's lymphoma	7 (3%)	8 (4%)
CMV	0	35 (16%)
Cryptosporidiosis	0	6 (3%)
Candidiasis	0	6 (3%)
Kaposi's sarcoma	0	2 (1%)
Cirrhosis	6 (3%)	0
Pulmonary hemorrhage	1 (0.5%)	0
Cerebral malaria	1 (0.5%)	0
Pyogenic meningitis	1 (0.5%)	0
Bacterial peritonitis	1 (0.5%)	0
Acute pyelonephritis	1 (0.5%)	0
Amyloidosis	1 (0.5%)	0
Sq. cell carcinoma lung	1 (0.5%)	0

CMV, cryptosporidiosis, candidiasis, and Kaposi's sarcoma contributed to death, other infectious diseases identified were aspergillosis of lung 6 (cases, 3%) and intestinal strongyloidiasis (3 cases, 1%).

**Table 3:** Comparison of different autopsy studies in HIV/AIDS.

Reference No.	[55]	[62]	[67]	[68]	[72]	[75]	[80]	[81]	[85]	[90]	Present study
Year and country	1988	1992	1993	1994	1996	1998	2000	2002	2003	2008	2010
	USA	Mexico	Africa	USA	USA	USA	USA	Africa	Russia	Brazil	India
No. of Autopsies	164	177	247	565	168	233	390	104	321	250	236
M. TB	—	44	94	76	45	13	—	42	45	36	152
		(25%)	(38%)	(13%)	(27%)	(5%)		(40%)	(14%)	(14%)	(68%)
MAI	29	—	—	104	26	31	85	—	—	15	—
	(18%)			(18%)	(15%)	(13%)	(21%)			(6%)	
Cryp	11	18	8	78	09	23	—	—	—	9	18
	(8%)	(10%)	(3%)	(14%)	(5%)	(10%)				(3%)	(8%)
Toxo	14	34	37	51	05	—	11	—	22	18	15
	(9%)	(19%)	(15%)	(9%)	(3%)		(3%)		(7%)	(7%)	(6%)
CMV	81	122	45	286	36	40	195	—	61	33	35
	(49%)	(69%)	(18%)	(51%)	(21%)	(17%)	(50%)		(19%)	(13%)	(16%)
PCP	99	42	7	308	55	56	100	11	—	68	11
	(60%)	(24%)	(3%)	(55%)	(33%)	(24%)	(26%)	(11%)		(27%)	(5%)
Cand	74	—	—	240	23	6	—	—	—	—	6
	(45%)			(42%)	(14%)	(2%)					(3%)
Histo	6	—	5	13	03	2	—	—	—	3	—
	(4%)		(2%)	(2%)	(2%)	(1%)				(1%)	
Crysp	7	—	7	—	—	—	—	—	—	—	6
	(4%)		(3%)								(3%)
H. Simplex	17	—	5	92	—	—	—	—	—	—	—
	(10%)		(2%)	(16%)							
H. Zoster	—	—	5	—	—	—	—	—	—	—	—
			(2%)								
Asperg	—	—	—	—	09	2	—	—	—	—	6
					(5%)	(1%)					(3%)
Bact Pneum	—	—	74	—		98	149	24	26	91	48
			(30%)			(42%)	(41%)	(23%)	(8%)	(36%)	(21%)
Nocardia	—	—	10	—	—	—	—	—	—	—	—
			(4%)								
KS	45	53	22	138	—	19	41	11	32	11	2
	(27%)	(30%)	(9%)	(24%)		(8%)	(10%)	(11%)	(10%)	(4%)	(1%)
NHL	17	16	7	81	—	3	20	—	—	5	8
	(10%)	(9%)	(3%)	(14%)		(1%)	(5%)			(2%)	(3%)
PML	—	—	—	—	—	—	12	—	—	—	—
							(3%)				
Str sterco	—	—	—	—	—	—	—	—	—	—	3
											(1%)
Discrepancy		—		—	—	—	—	—	—	—	42%

KS: Kaposi's sarcoma, Toxo: Toxoplasmosis, Cryp: Cryptococcosis, Cand: Candidiasis, Crsp: cryptosporidiosis, Histo: Histoplasmosis, Asperg: Aspergilosis, bact Pneum: bacterial pneumonia, NHL: Non-Hodgkin's lymphoma, PML: Progressive multifocal leucoencephalopathy, Str sterco: Strongiloides stercoralis.
